# Endoscopic treatment for calcific tendinitis of the gluteus medius: A case report and review

**DOI:** 10.3389/fsurg.2022.917027

**Published:** 2022-10-24

**Authors:** Chen Jian, Wang Dan, Wang Gangliang

**Affiliations:** ^1^ Department of Orthopedics, Sir Run Run Shaw Hospital, Hangzhou, Zhejiang, China; ^2^ Department of Orthopedics, Tonglu First People's Hospital, Hangzhou, China

**Keywords:** calcific tendinitis, gluteus medius, hip pain, minimally invasive (MI), endoscopic treatment

## Abstract

Tendon calcification is a common disease, with the shoulder rotator cuff being the most common site. However, calcific tendinitis of the gluteus medius has rarely been reported. This study reports the case of a 64-year-old woman diagnosed with calcific tendinitis of the gluteus medius and experiencing right lateral hip pain with no apparent trigger. After unsuccessful conservative treatment, hip endoscopy was performed on this patient, allowing for a clear view of a “toothpaste-like” lesion in the gluteus medius tendon. A shaver was used to remove the lesion. After 8 weeks and 14 months of follow-up, the patient could return to regular daily and social activities. This study shows that endoscopic surgery can lead to effective, rapid recovery and minimally invasive clinical outcomes in patients with tendon calcification near the hip joint.

## Introduction

Calcific tendinitis is the deposition of calcium hydroxyapatite crystals in periarticular muscular attachments (1). The etiology of calcific tendinitis is unclear, and the suggested causes include hereditary, metabolic, post-traumatic, and postoperative conditions (2).

Several tendons can become calcified, including those in the shoulder rotator cuffs, near the hip joints, and in hands and wrists (1, 2). However, calcific tendinitis of the gluteus medius, first described by Goldenberg and Leventhal in 1936 (3), is relatively uncommon and sporadically reported (4–8).

Generally, conservative methods for calcific tendinitis are satisfactory, including physiotherapy, non-steroidal anti-inflammatory drugs, local glucocorticoid injection, extracorporeal shock wave therapy, and small needle scalpel therapy (7). However, surgical treatment is usually performed in refractory cases.

Hip endoscopy is minimally invasive and entirely extra-articular, which can help avoid incising the hip capsule fibers, which causes hip joint instability. However, this procedure has been reported only in a few calcific tendinitis cases (2, 6, 9–11). Herein, we report the case of a patient with rare calcific tendinitis of the gluteus medius.

## Case report

The case of a 64-year-old woman with a history of diabetes mellitus and hypertension is reported here. The patient presented with a 2-year history of right lateral hip pain with no apparent trigger. The pain was aggravated after 10 min of walking and relieved after a break. The pain was predominantly located in the greater trochanter and did not radiate to the lower back or lower extremities. The patient did not experience fever, chills, or limb weakness. However, hip pain was increasingly severe over the past year, hindering hip motion and interfering with sleep. The patient unsuccessfully attempted conservative treatment with oral non-steroidal anti-inflammatory drugs (NSAIDs) and traditional Chinese medicine.

The physical examination revealed an antalgic gait. The Trendelenburg and FABER (flexion abduction external rotation) tests were positive. Moderate tenderness upon palpation of the greater trochanter was experienced. The range of motion was limited compared with the left side. The range of flexion was 90°, extension was 10°, abduction was 25°, and adduction was 20°. The legs were lengthened equally, and neurological examination showed no abnormalities. The initial Harris hip score was 57.45, and the visual analog scale (VAS) score for hip pain in the resting state was 4.

**Figure 1:**
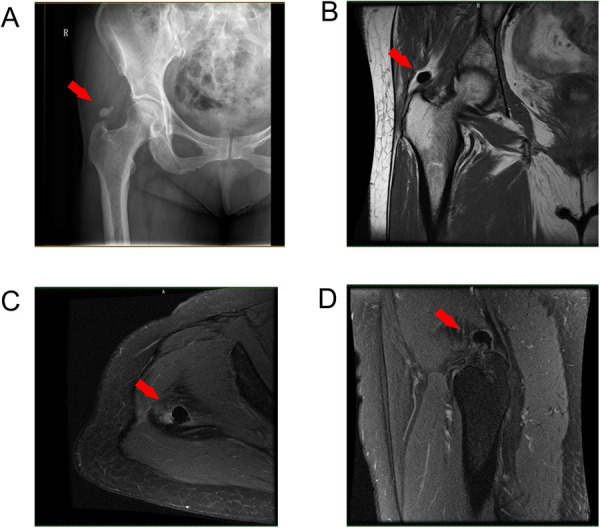
(**A**) Preoperative plain radiograph, (**B**) T2 coronal, (**C**) T1 horizontal, and (**D**) T1 coronal. View of MRI showing the calcification around the tip of the right greater trochanter (marked with arrows).

Plain radiography showed a 1.0 × 2.0 cm diameter calcific deposit superomedial to the greater trochanter; this corresponded to the tendinous insertion of the gluteus medius (Figure 1A). Magnetic resonance imaging (MRI) showed evidence of calcific tendinitis (Figure 1B–D); calcific deposits were hypointense on T1- and T2-weighted images. The sedimentation rate and white blood cell count were within normal ranges.

**Figure 2:**
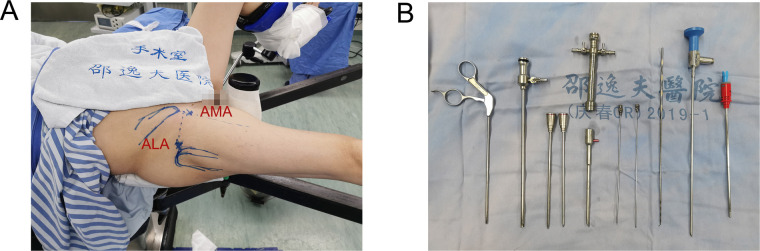
(**A**) The patient was placed supine on a traction table; an anterolateral approach (ALA) was approximately 1 cm superior and anterior to the anterior edge of the greater trochanter; an anteromedial approach (AMA) was performed at the junction of a sagittal line drawn from the anterior superior iliac spine and a horizontal line drawn from ALA (**B**) surgical instruments used in hip endoscopic surgery.

As conservative treatment was unsuccessful, hip endoscopy was performed (6, 9, 12). The patient was placed supine on a traction table. A sufficient amount of traction was applied to the operative hip to provide a 10–12 mm distraction of the hip joint. Standard anterolateral, mid-anterior, and distal anterolateral portals were used. An anterolateral approach (ALA) was first performed under fluoroscopic control, which was approximately 1 cm superior and anterior to the anterior edge of the greater trochanter. Next, an anteromedial approach (AMA) was established under endoscopy surveillance (Figure 2A). A 30° hip scope was inserted to visualize the gluteus medius tendon and the tip of the greater trochanter (Figure 2B).

**Figure 3:**
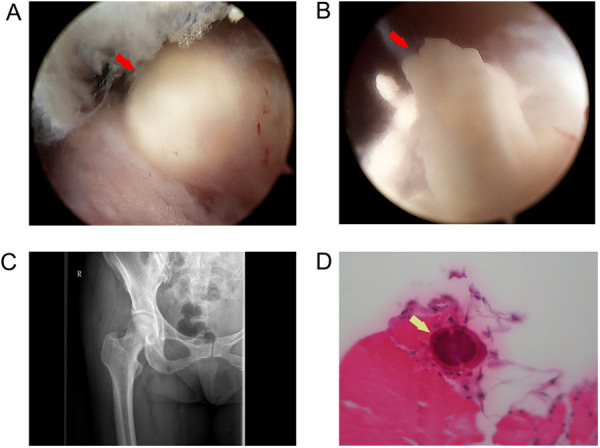
(**A**) Endoscopic view of a calcified deposit, (**B**) Endoscopic view showing a soft white toothpaste-like material in the deposit, (**C**) postoperative plain radiograph view showing complete removal of the calcific deposit, and (**D**) hydroxyapatite crystals in the specimen confirmed by a pathologic examination (marked with arrows).

Endoscopic imaging confirmed the radio-opacity on plain radiography. A soft white toothpaste-like material was observed when the degenerated gluteus medius tendon was debrided (Figure 3A,B). A motorized shaver was used to remove the material and debride the degenerated tendons. Postoperative radiography revealed that no calcification remained (Figure 3C). The specimen was sent for pathologic examination, which confirmed that the calcified tendon consisted of hydroxyapatite crystals and proliferative tendon fibrous tissue (Figure 3D).

The patient was mobilized and allowed to bear weight as tolerated. In addition, straight-leg raise exercises were started on postoperative day one and resisted knee extension strengthening exercises continued for 6 weeks. The Trendelenburg test and Patrick sign were negative at 8 weeks, and no restrictions were imposed on activities due to symptoms. The last recorded Harris hip score was 92.45, and the VAS score was 1 at 14 months after surgery.

## Discussion

In the present case, the patient was diagnosed with calcific tendinitis of the gluteus medius. Deposing focal apatite crystals in the tendon can result in acute or chronic hip pain during daily activities; joint movements may aggravate these symptoms (13). The presenting symptoms, in this case, were chronic pain in the posterolateral region of the right hip and right hip movement limitations.

The most common site of calcific tendinitis is the shoulder, occurring in approximately 3% of adults (1); other sites include the joint tendons such as wrists (14) or knees (15–17). In addition, calcific tendinitis has been reported in tendons near the hip joint, such as the gluteus minimus (6), gluteus medius (18), gluteus maximus (12, 19), gemellus superior, gemellus inferior (2), and rectus femoris (9).

The gluteus medius is located on the lateral side of the iliac wing; it is the main abductor muscle in the hip joint. It plays an important role in both standing and walking. Degeneration and strain of the tendon, in this case, are possible causes of the patient’s calcific tendinitis.

The calcium around the greater trochanter can cause mild to severe pain according to different calcific stages as described in the Uhthoff HK (20). Stage 1: Precalcific phase, fibrocartilaginous transformation within tendon fibers, which is asymptomatic. Stage 2: Formative phase, calcifications formed, usually causing subacute mild pain. Stage 3: Resorptive phase, the tendon develops increased vasculature and calcium deposits are removed by phagocytes; severe acute pain that can be highly disabling and unresponsive to common analgesics is experienced. Stage 4: Postcalcific phase, self-healing, and repair of the tendon fibers over several months, associated with pain and restricted function. Thus, different degrees of pain exist in stages 2–4, which explains why, in some cases, calcium around the greater trochanter can be found incidentally but is not clinically symptomatic.

The treatment for calcific tendinitis of the gluteus medius is summarized in Table 1. Surgical treatment can be adopted in patients with failed conservative treatments or severe symptoms. However, the use of endoscopy to treat the disease is not widespread, as tendon calcification of the hip joint is rare and sporadic, and endoscopic treatment has been reported only in a few cases (2, 6, 9–11). Comba et al. (10). described the surgical technique of endoscopic surgical removal in patients with calcific tendinitis of the rectus femoris. Kandemir et al. (6). and Su et al. (12). described the endoscopic treatment of the gluteus medius and maximus calcified tendinitis and considered it an effective treatment.

**Table 1:** Review of articles regarding the treatment of calcific tendinitis of the gluteus medius.

No.	First author	Patient No.	Treatment	Follow-up	Outcomes	Reference
1	Yang I (4)	1	Direct injection with local anesthetic and steroids at the gluteus lesion and anti-inflammatory agents	24 months	Symptom-free	*Skeletal Radiol* 2002
2	Kandemir U (6)	1	Endoscopic treatment	Three months	symptom-free	*Arthroscopy* 2003
3	Sakai T (5)	1	Non-steroidal anti-inflammatory drugs (NSAIDs)	Two years	Clinical symptoms had not recurred	*J Orthop Sci* 2004
4	Lin W (18)	1	Acupuncture and small needle scalpel therapy	Six months	Satisfied with the condition	*Acupunct Med* 2012
5	Almedghio S (8)	2	Analgesia and NSAIDs	Three weeks; N/A	Pain-free; symptom complete resolution	*J Orthop Case Rep* 2014
6	Vereecke E (21)	1	Ultrasound-guided needle lavage and injection of anesthetic/corticosteroid	Within a few days	Symptoms resolved	*J Belg Soc Radiol* 2015
7	Jo H (7)	1	Ultrasound-guided barbotage of the calcification	Six months	Remained pain-free	*Ann Rehabil Med* 2016

In our case, endoscopic surgery was used to treat the patient, which led to effective, rapid recovery and a minimally invasive clinical outcome. Therefore, given the patient's outcome to date, endoscopic treatment of calcific tendinitis of the gluteus medius may be an appropriate treatment option.

Clinically, tendon calcification of the hip joint usually presents pain and tenderness around the hip with movement limitations. These atypical symptoms make it possible to misdiagnose the disease as osteochondroma, gout, ossifying myositis, heterotopic calcification, or lumbar spinal disease.

A thorough medical history and physical examination are essential for diagnosis. For example, patients with heterotopic calcification and myositis ossificans often have a history of trauma (11), while those with gout often have a high uric acid level. Imaging techniques, such as ultrasound, computed tomography (CT) (19, 22), or MRI (5), should be used in cases of diagnostic uncertainty. CT may help evaluate osseous involvement, while MRI can demonstrate tissue edema and rule out bone tumors. In addition, the typical presenting symptoms of lateral hip pain may occasionally mimic lumbar radiculopathy and vice versa. Therefore, obtaining a lumbar MRI may be warranted in select patients (2, 7).

## Conclusion

We report a well-documented case of rare calcific tendinitis of the gluteus medius. Endoscopic surgery is considered a feasible and effective option for patients with hip calcific tendinitis who do not respond to conservative treatment, with few risks, rapid recovery, and satisfactory outcomes. However, a longer follow-up time and a large patient sample size are needed to draw reliable conclusions.

## Data Availability

The original contributions presented in the study are included in the article/Supplementary Material, further inquiries can be directed to the corresponding author/s.
